# Critical role of FGF21 in diabetic kidney disease: from energy metabolism to innate immunity

**DOI:** 10.3389/fimmu.2024.1333429

**Published:** 2024-01-19

**Authors:** Yingnan Liang, Qi Chen, Yue Chang, Junsong Han, Jiaxin Yan, Zhenjie Chen, Jingwei Zhou

**Affiliations:** Department of Nephrology, Dongzhimen Hospital, Beijing University of Chinese Medicine, Beijing, China

**Keywords:** diabetic kidney disease, energy metabolism, fibroblast growth factor 21, innate immunity, inflammation

## Abstract

Diabetic kidney disease (DKD) stands as the predominant cause of chronic kidney disease (CKD) on a global scale, with its incidence witnessing a consistent annual rise, thereby imposing a substantial burden on public health. The pathogenesis of DKD is primarily rooted in metabolic disorders and inflammation. Recent years have seen a surge in studies highlighting the regulatory impact of energy metabolism on innate immunity, forging a significant area of research interest. Within this context, fibroblast growth factor 21 (FGF21), recognized as an energy metabolism regulator, assumes a pivotal role. Beyond its role in maintaining glucose and lipid metabolism homeostasis, FGF21 exerts regulatory influence on innate immunity, concurrently inhibiting inflammation and fibrosis. Serving as a nexus between energy metabolism and innate immunity, FGF21 has evolved into a therapeutic target for diabetes, nonalcoholic steatohepatitis, and cardiovascular diseases. While the relationship between FGF21 and DKD has garnered increased attention in recent studies, a comprehensive exploration of this association has yet to be systematically addressed. This paper seeks to fill this gap by summarizing the mechanisms through which FGF21 operates in DKD, encompassing facets of energy metabolism and innate immunity. Additionally, we aim to assess the diagnostic and prognostic value of FGF21 in DKD and explore its potential role as a treatment modality for the condition.

## 1 Introduction

Diabetic kidney disease (DKD) stands as a prevalent complication of diabetes mellitus (DM), estimated to affect 50% patients with DM globally (1). The prevalence of DM among adults aged 20 to 79 over the world is anticipated to reach 12.2% (783.2 million) by 2045 (2). Concurrently, as the prevalence of DM continues to rise, DKD has emerged as the predominant cause of chronic kidney disease on a global scale, imposing a substantial economic burden. The natural progression of DKD encompasses glomerular hyperfiltration, progressive albuminuria, a gradual decline in the estimated glomerular filtration rate (eGFR), ultimately culminating in end-stage renal disease. It is imperative to elucidate the pathogenesis of DKD and institute preventive measures at an early stage (3). DKD is usually a clinical diagnosis made based on the presence of albuminuria and/or reduced eGFR in the absence of signs or symptoms of other primary causes of kidney damage (4). Under comprehensive and multifactorial interventions, the clinical manifestations of DKD have shown an increasingly complex heterogeneity, which can be characterized by both a clinical phenotype with no proteinuria or with the appearance of proteinuric remission, and a clinical phenotype with a rapid decline in eGFR (5). Notably, studies have revealed that even in DM patients exhibiting normal urinary protein levels, there exists a greater than 50% probability of grade IIa or more severe glomerulopathy (6). Therefore, more sensitive biomarkers are needed for early diagnosis and prediction of DKD. In recent years, the completion of several phase 3 clinical trials has ushered in a transformative era in the management of DKD, prompting the expeditious revision of clinical guidelines (7, 8). However, a residual risk of DKD progression persists, underscoring the need for novel treatments (9).

Fibroblast growth factor 21 (FGF21) emerges as a stress-inducible hormone, operating in concert with FGF receptor 1 (FGFR1) and β-klotho as a heterodimeric receptor complexz. Its regulatory prowess extends to energy balance, glucose, and lipid homeostasis, yielding significant benefits such as the reduction of fat mass and mitigation of hyperglycemia, insulin resistance, dyslipidemia, cardiovascular disease, and non-alcoholic steatohepatitis (10). Energy metabolism plays a key role in innate immune regulation. Lipid metabolism and the signaling pathways defining macrophage functions are intertwined, enabling coordinated regulation of macrophage biology (11). Immune system cells that are activated, split, and differentiated during an immune response depend on the metabolic reprogramming of both catabolic and anabolic pathways to produce metabolites that play crucial roles in regulating the response in addition to producing energy in the form of ATP (12). FGF21, beyond its role as a metabolic regulator, assumes the mantle of an innate immunity regulator, exerting anti-inflammatory effects (13).

DKD is instigated by disruptions in glucose metabolism associated with DM. These disruptions subsequently activate various metabolic, hemodynamic, inflammatory, and fibrotic processes that collectively contribute to the progression of the disease. The intertwining of energy metabolism and innate immunity plays a crucial role in the advancement of DKD (14). Serving as a focal point bridging energy metabolism and compromised innate immunity, FGF21 holds promise as a potential target for the diagnosis and treatment of DKD.As research interest burgeons around the multifaceted functions and pharmacological potential of FGF21, it has become a focal point in recent years (15). However, despite the increasing attention, a systematic exploration of the relationship between FGF21 and DKD remains lacking. To address this gap, we conducted a comprehensive review of relevant studies, elucidating the mechanisms and application value of FGF21 in the context of DKD.

## 2 Energy metabolism and innate immunity in DKD

**Figure 1:**
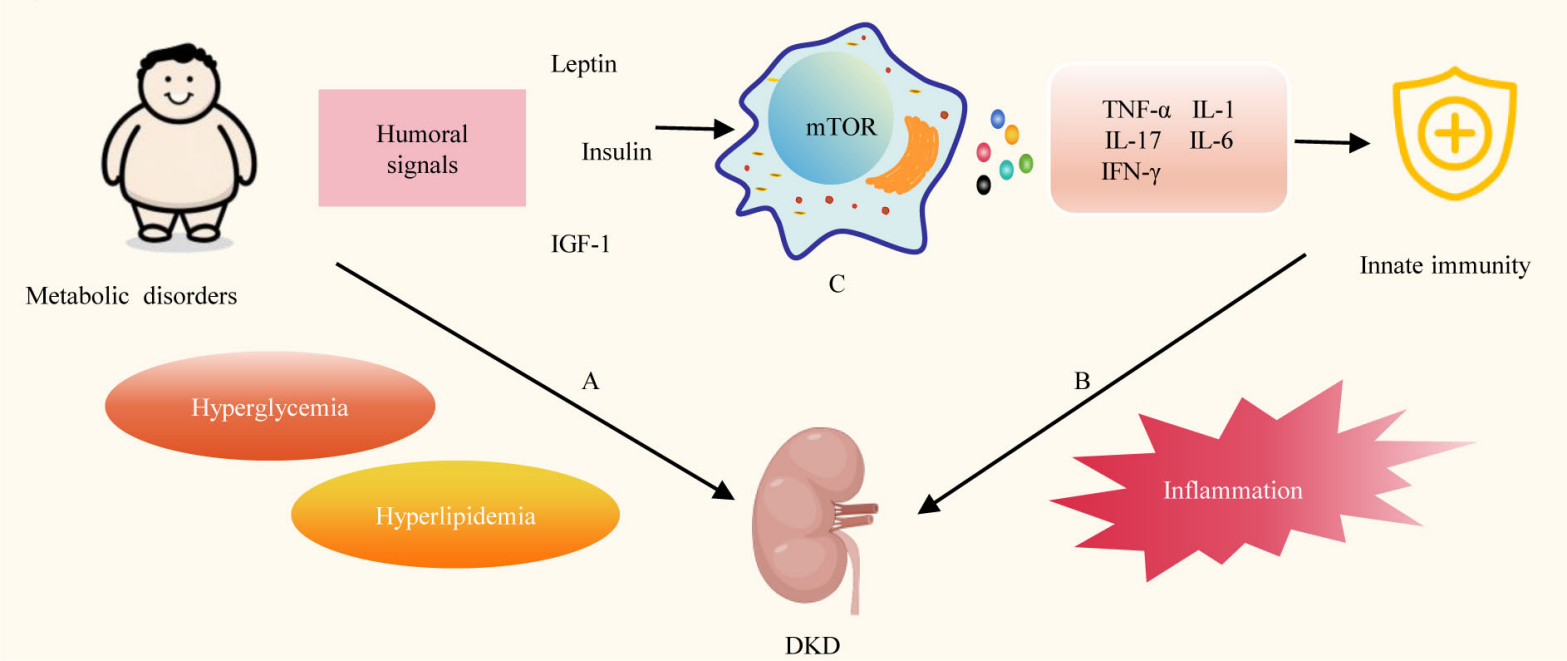
Energy metabolism and Innate immunity in DKD. **(A)** Disturbed energy metabolism is one of the main pathogenic mechanisms of DKD. **(B)** Innate immunity plays a key role in the progression of DKD. **(C)** Metabolic disorders can affect innate immune cell metabolism, signaling and the release of various cytokines. TGF-1, Insulin-like growth factor-1; mTOR, mechanistic target of rapamycin; TNF-α, tumor necrosis factor-alpha; IL-1, interleukin-1; IL-6, interleukin-6; IL-17, interleukin-17; IFN-Y, interferon-gamma.

Disturbed energy metabolism is one of the main pathogenic mechanisms of DKD (**Figure 1A**). DM is associated with the dysregulation of various metabolites, such as glucose, insulin, and lipids, contributing to the progression of DKD (16). DKD usually develops in genetically susceptible individuals due to poor metabolic (blood sugar) control. Hyperglycemia alters endothelial and podocyte metabolism, overburdening proximal tubular cells, which are a major source of cellular stress in the kidney. Lipid metabolites may also predict DKD progression (17). DM inflicts direct injury upon renal tubules, resulting in mitochondrial dysfunction. This dysfunction encompasses reduced bioenergetics, an accumulation of mitochondrial reactive oxygen species (mtROSs), impaired mitophagy, and dynamic abnormalities. These dysfunctions, in turn, initiate a cascade of abnormalities related to metabolism (18).

Concurrently, innate immunity plays a pivotal role in the progression of DKD. (**Figure 1B**) Multiple innate immune cells, molecules, and signaling pathways are involved in the pathogenesis of DKD (19). Innate immune malfunction in DKD patients leads to inflammation. Inflammation is triggered in reaction to damaging circumstances to preserve tissue integrity and homeostasis. Nevertheless, persistent stimulation of the inflammatory response results in unintended harmful consequences (20). Toll-like receptors (TLRs) emerge as a dynamic receptor family adept at recognizing both pathogen-associated molecular patterns and damage-associated molecular patterns in the context of DM. This recognition process facilitates the activation of leukocytes and intrinsic renal cells in DKD, thereby initiating the proinflammatory cascade during DKD (21). A variety of proinflammatory molecules, chemotactic factors, and adhesion molecules, through multiple inflammatory pathways involving mitogen-activated protein kinases (MAPK), signal transducer and activator of transcription (STAT), and protein kinase C (PKC), induce interactions among intrinsic renal cells and various innate immune cells (macrophages, dendritic cells). These interactions contribute to the progression of DKD (22). Additionally, the complement system, as a crucial component of the innate immune system, swiftly generates a substantial quantity of protein fragments upon activation. These fragments serve as effective mediators for inflammation, vascular activity, and metabolic responses. They also contribute to the progression of DKD (23). Insights from preclinical studies suggest that targeting these innate immune pathways holds promise for developing novel therapies for DKD (24).

Energy metabolism plays a key role in the regulation of innate immunity (**Figure 1C**) Changes in cell metabolism affect the activity of immune cells, and the function of immune cells in turn determines the metabolic state of cells. Immune cells require a lot of energy to activate. Different methods are used by immune cells to produce this energy. Energy is produced by boosting glycolysis in pro-inflammatory cells like M1 macrophages, and by enhancing mitochondrial activity and beta-oxidation in regulatory cells such as M2 macrophages (25). Overnutrition is linked to low-grade, chronic inflammation that raises the risk of metabolic and cardiovascular disease, promotes autoreactivity, and compromises protective immunity, while undernutrition is linked to immunosuppression, which increases susceptibility to infection and protection against various types of autoimmune disease (26). DM is a chronic metabolic disorder, and insufficient insulin secretion and insufficient insulin action are the two main reasons for the development of DM (27). Metabolic disorders induce the expression of nutritionally and metabolically related growth factors, such as leptin, insulin, and insulin-like growth factor 1 (IGF-1), which activate mechanistic target of rapamycin (mTOR) signaling in immune cells, thereby affecting systemic and intracellular immune metabolism, and consequently inflammation. Further promotes the production of inflammatory cytokines such as interleukin-1 (IL-1), tumor necrosis factor-alpha (TNF-α), interleukin-6 (IL-6), interleukin-17 (IL-17), and interferon-gamma (28). Proinflammatory cytokine production and low-grade inflammation both locally and systemically are linked to the onset and advancement of DKD (29).

## 3 FGF21 is a bridge between energy metabolism and innate immunity

### 3.1 FGF21 regulates energy metabolism in DM patients

**Figure 2:**
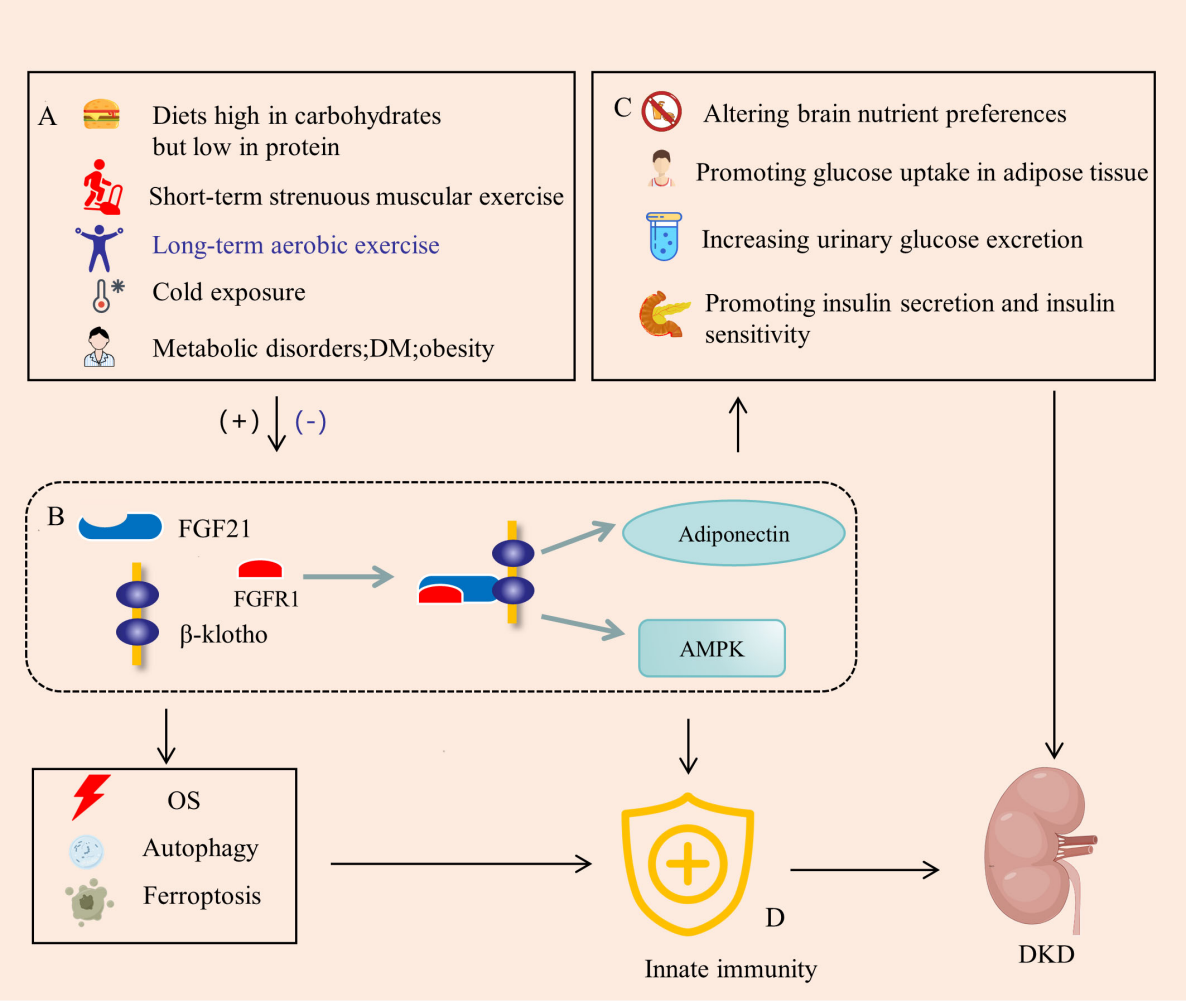
FGF21 is a bridge between energy metabolism and innate immunity. **(A)** FGF21 is induced by diet, exercise, cold, and metabolic disorders, significantly up-regulated in DM environments. **(B)** FGF21 binds to FGFR1 and β-klotho to form a complex that activates AMPK signaling in target tissues and promotes adiponectin expression. **(C)** FGF21 regulates metabolism by altering brain nutrient preferences, promoting glucose uptake in adipose tissue, increasing urinary glucose excretion, and promoting insulin secretion and insulin sensitivity. **(D)** FGF21 can also directly regulate innate immune signals and pathways, or indirectly regulate innate immunity through OS, autophagy and ferroptosis. DM, diabites mellitus; AMPK, AMP-activated protein kinase; OS, oxidative stress. (+), Upregulation; (-), Downregulation.

FGF21, a peptide hormone synthesized by various organs, plays a crucial role in regulating energy homeostasis. It functions as an autocrine, paracrine, and endocrine factor, exerting diverse metabolic effects on several target organs (30). The molecular mechanism of FGF21 signaling is complex and involves several FGF receptors (FGFRs), FGF21 binds to FGFR with very low affinity, and effective binding and signaling requires interaction with the co-receptor β-klotho (31). FGF21 production is influenced by factors such as diet, exercise, and environmental temperature (32) (**Figure 2A**). In a study employing a whole-room indirect calorimeter, researchers assessed alterations in plasma FGF21 concentrations in 64 healthy individuals with well-regulated glucose levels after a 24-hour period of exposure to seven food treatments with varying macronutrient contents. Notably, it was only after the consumption of two low-protein (3%) overfeeding diets—one rich in carbohydrates (75%) and the other in fat (46%)—that plasma FGF21 concentrations consistently surged by threefold. Larger increments in FGF21 were positively correlated with greater enhancements in 24-hour energy expenditure. These findings underscore that diets characterized by high carbohydrate content but low protein content substantially elevate circulating FGF21 levels (33). The impact of exercise on FGF21 is contingent upon the type of exercise performed. Short-term strenuous muscular exercise has been associated with an elevation in serum FGF21 levels, primarily attributed to the stimulation of skeletal muscle production. In contrast, long-term aerobic exercise has been shown to significantly decrease serum FGF21 concentrations (34). A separate clinical investigation has revealed that exposure to mild cold results in elevated levels of circulating FGF21, thereby anticipating heightened lipolysis and cold-induced thermogenesis. Subtle reductions in ambient temperature have proven effective in regulating FGF21 circadian rhythms in humans, potentially acting as a mediator for cold-induced metabolic alterations analogous to those observed in animals (35). Elevated levels of circulating FGF21 are observed in various metabolic disorders, including DM, obesity, and cardiovascular disease. This seemingly paradoxical occurrence is commonly characterized as a state of “FGF21 resistance” or viewed as a compensatory protective response to metabolic stress (36, 37). A clinical investigation assessed circulating FGF21 levels in 2066 hospitalized patients with DM, revealing that individuals in the low urinary glucose excretion group exhibited higher body mass index (BMI) and serum FGF21 levels. Additionally, lower urinary glucose excretion was found to be associated with increased insulin resistance in patients with type 2 DM (T2DM) (38).

FGF21 exerts its influence through various downstream factors (**Figure 2B**). Mouse experiments have illustrated that FGF21 treatment enhances both the expression and secretion of adiponectin in adipocytes, consequently elevating serum adiponectin levels in mice. Notably, the therapeutic benefits of FGF21 are compromised in Adiponectin Knockout (KO) mice, highlighting the integral role of adiponectin in mediating the multiple therapeutic effects of FGF21. Consequently, FGF21 actions within local adipocytes are intricately linked to the liver and skeletal muscle through adiponectin, thereby facilitating the systemic effects of FGF21 on insulin sensitivity and energy metabolism (39). However, in obese animals and human subjects, there is a paradoxical scenario where circulating FGF21 levels are elevated, while plasma adiponectin concentrations are diminished, potentially attributed to FGF21 resistance (40). Additionally, the AMP-activated protein kinase (AMPK), a crucial player in preserving tissue integrity and energy balance, is implicated in this process. Endocrine FGF21 signaling has been demonstrated to stimulate the AMPK system, either directly through FGFR1/β-klotho signaling or indirectly by promoting the release of corticosteroids and adiponectin. These released factors, in turn, activate AMPK signaling in the target tissues, contributing to the intricate regulatory network of FGF21 in energy metabolism (41).

In the DM, the up-regulation of FGF21 orchestrates the regulation of blood glucose and metabolism through a comprehensive fourfold action (**Figure 2C**). Firstly, FGF21 exerts its influence on the brain to homeostatically modify macronutrient preference. Notably, exogenous FGF21 therapy induces a reduction in the consumption of alcohol and sweets, concomitant with an augmentation in protein intake (42). Secondly, FGF21 is locally released from adipose tissue following activation of the β-adrenergic receptor, subsequently enhancing glucose uptake in adipocytes (43). The application of a half-life extended form of FGF21 (FGF21-PEG) has been demonstrated to normalize plasma glucose levels in streptozotocin-treated mice, a model of type 1 diabetes mellitus (T1DM), without reinstating pancreatic β-cell function. This study underscores that insulin-independent glucose uptake in adipocytes persists even in the presence of insulin receptor antagonists (44). Thirdly, administration of FGF21 to mice with both T2DM and T1DM led to a dose-dependent decrease in the kidney’s glucose transport maximum, accompanied by an increase in urine glucose excretion. Experimental findings in mice indicate that FGF21 mitigates hyperglycemia, in part, by reducing renal glucose reabsorption through the peroxisome proliferator-activated receptor-δ (PPARδ)-mediated sodium-glucose cotransporter 2 (SGLT2) pathway (45). Fourthly, the absence of FGF21 (FGF21 KO) exacerbated palmitate-induced islet β-cell failure and suppression of glucose-stimulated insulin secretion (GSIS). In db/db mice, overexpression of pancreatic FGF21 markedly improved islet morphology, elevated GSIS, decreased β-cell apoptosis, and increased insulin expression. Pancreatic FGF21 in T2DM mice induced phosphatidylinositol 3-kinase (PI3K)/Akt signaling-dependent insulin expression and secretion (46). FGF21 demonstrates protective effects against lipotoxicity-induced β-cell dysfunction and apoptosis by down-regulating islet cell lipid accumulation and reducing cell death under lipotoxic conditions, likely through the activation of AMPK-acetyl-CoA carboxylase (ACC) and PPARδ/γ signaling (47). Moreover, experiments have indicated that FGF21 represses mammalian target of rapamycin complex 1 (mTORC1), thereby improving insulin sensitivity and glycogen storage in a hepatocyte-autonomous manner (48). Therapeutic administration of FGF-21 to both ob/ob and db/db mice brought their plasma glucose and triglyceride levels close to normal, with effects persisting for a minimum of twenty-four hours after cessation of FGF-21 treatment. Notably, FGF-21 did not induce hypoglycemia or weight gain (49). The FGF21-mediated regulation of blood glucose and metabolism has been demonstrated to contribute to the delay in the progression of DKD (50).

### 3.2 FGF21 regulates innate immunity in DM patients

An expanding body of research substantiates the involvement of FGF21 in the regulation of innate immunity(**Figure 2D**). Endothelial dysfunction, a precursor to proteinuria, glomerular sclerosis, and interstitial fibrosis, contributes to the progression of DKD (51). FGF21 plays a pivotal role in enhancing endothelial cell function through the modulation of innate immunity and the inhibition of inflammatory pathways. In a cellular experiment utilizing varying concentrations of recombinant FGF21 to treat human umbilical vascular endothelial cells (HUVECs), a notable improvement in glycolysis, increased nitric oxide release, and cellular protection against oxidative damage induced by H_2_O_2_ were observed at an optimal FGF21 concentration of 400 pg/mL. Subsequent to FGF21 treatment, the majority of upregulated genes were enriched in metabolic pathways, while downregulated genes were associated with signaling pathways linked to apoptosis and inflammation (52). Another cellular experiment suggested that FGF21 may mitigate uric acid (UA)-induced endoplasmic reticulum (ER) stress, inflammation, and vascular endothelial cell dysfunction by activating sirtuin 1 (Sirt1) (53). By targeting PPARγ, miR-27b was found to trigger the nuclear factor-κB (NF-κB) signaling pathway and the expression of inflammatory factors, including IL-1β, IL-6, and TNF-α. FGF21 alleviated hypoxia-induced dysfunction and inflammation in human pulmonary arterial endothelial cells by inhibiting miR-27b expression and consequently promoting PPARγ expression (54). Moreover, studies indicated that the deletion of FGF21 in mice exacerbated deoxycorticosterone acetate (DOCA)-salt-induced nephropathy. Supplementation with recombinant human FGF21 (RhFGF21) restored renal damage caused by DOCA and salt. Mechanistically, RhFGF21 activated AMPK, which suppressed NF-κB-regulated inflammation and nuclear factor erythroid 2-related factor 2 (NRF2)-mediated oxidative stress (OS) (55). FGF21 combined with insulin promotes the conversion of M1 macrophages to M2 macrophages to reduce inflammation in DN mice (56). Transcriptome analysis consequently demonstrated that, in FGF21 KO mice but not in WT mice, inflammation-related pathways were markedly enriched and elevated (57). In a mouse model of lipotoxicity and diabetes, FGF21 partially prevented renal injury induced by free fatty acids and diabetes by reducing renal lipid accumulation and inhibiting inflammation, oxidative stress (OS), and fibrosis (58).

Beyond its direct role in regulating innate immunity to suppress inflammation, FGF21 also indirectly influences innate immunity through oxidative stress (OS), autophagy, and ferroptosis, thereby contributing to the delayed progression of DKD. Prolonged OS has the potential to activate innate immunity through multiple transcription factors (59). FGF21, with its associations with genes such as NRF2, thioredoxin binding protein-2 (TBP-2), uncoupling protein 3 (UCP3), superoxide dismutase-2 (SOD2), extracellular signal-regulated kinase (ERK), and p38, and the identification of a crucial response element for activating transcription factor 4 (ATF4) involved in OS regulation, assumes a critical role as a regulator of the cellular response to OS (60). A cross-sectional study including 382 CKD patients showed an independent positive correlation between serum FGF21 and OS levels (61). FGF21 has demonstrated its capacity to inhibit vascular calcification, partially by restoring the antioxidant superoxide dismutase (SOD) levels and reducing vascular OS (62). The peroxisome proliferator-activated receptor alpha (PPARα) agonist, fenofibrate (FF), known for its efficacy in DKD, has been shown to prevent DKD development by mediating NRF2 pathway activation through FGF21 (63). Autophagy, a protective mechanism against various forms of renal inflammatory injury, is also under the regulation of FGF21. Through receptor for activated C kinase 1 (RACK1)-mediated AMPK activation and interaction with autophagy-related 5 (Atg5), FGF21 induces autophagy to enhance cholesterol efflux and minimize cholesterol accumulation in foam cells (64, 65). Epigenetically upregulating global autophagy-network genes, including transcription factor EB, Atg7, Atgl, and FGF21, Jumonji-D3 (JMJD3) demethylates histone H3K27-me3 in response to FGF21 stimulation, leading to autophagy-mediated lipid breakdown (66). FF has been shown to prevent type 1 diabetes-induced pathological and functional abnormalities of the heart by increasing FGF21, which may up-regulate Sirt1-mediated autophagy (67). Ferroptosis is a form of regulated necrosis, wherein excessive or deficient ferroptotic cell death is associated with a dysregulated immune response (68). FGF21 serves as a novel suppressor of ferroptosis. Both the administration of recombinant FGF21 and the overexpression of FGF21 have demonstrated significant protection against iron overload-induced damage to hepatocyte mitochondria, liver injury, and fibrosis by inhibiting ferroptosis. Conversely, the absence of FGF21 has been shown to exacerbate iron overload-induced ferroptosis (69).

## 4 FGF21 is a potential marker for the diagnosis and prognosis of DKD

**Figure 3:**
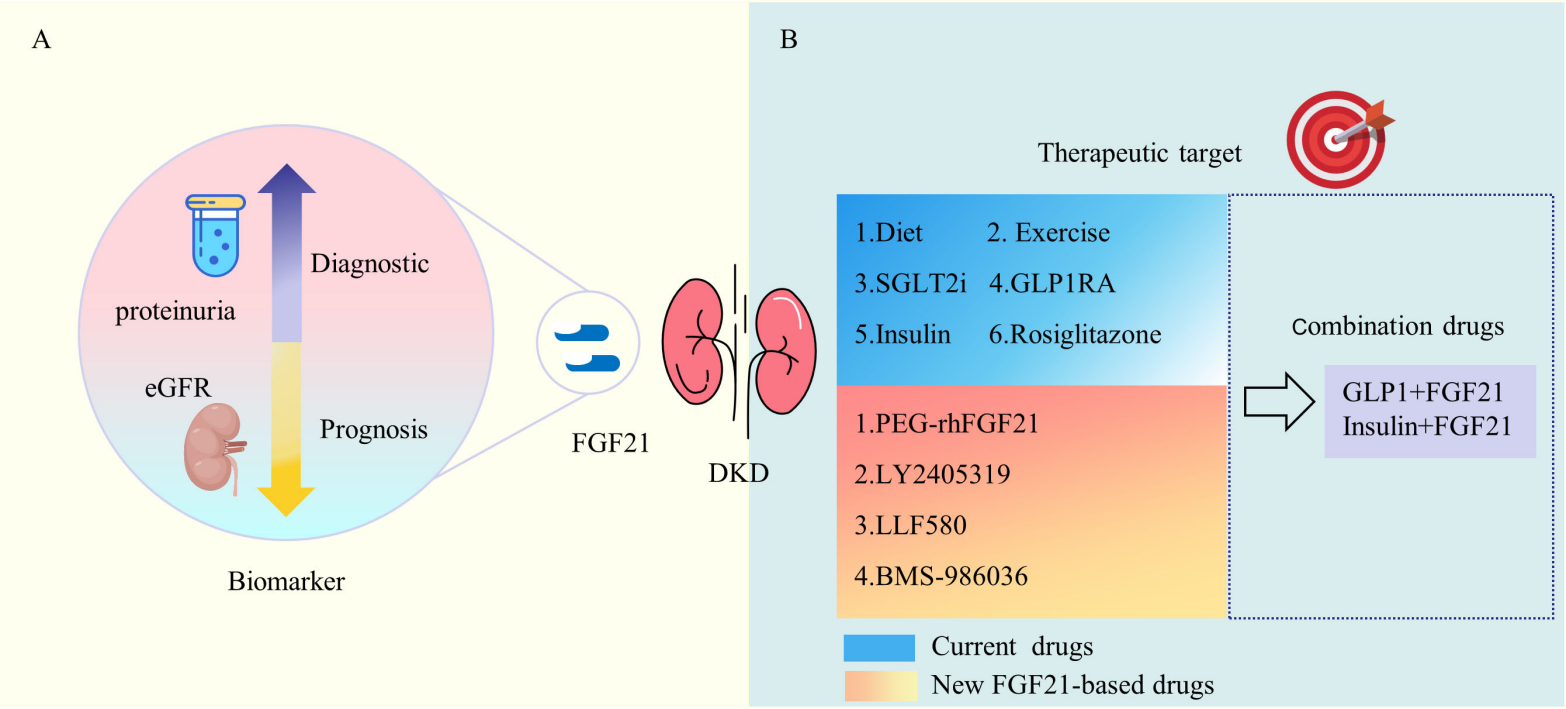
Clinical Use of FGF21 in DKD. **(A)** FGF21 is associated with DKD proteinuria and eGFR and is a marker for DKD diagnosis and prognosis. **(B)** Multiple current DKD treatments and medications including diet and exercise are associated with FGF21. A variety of new FGF21-based drugs show favorable metabolic modulation effects. eGFR, estimated glomerular filtration rate; SGLT2i, sodium-glucose cotransporter 2 inhibitor; GLP-IRA, glucagon-like peptide 1 receptor agonist; PEG-rhFGF21, PEGylated recombinant human FGF21.

FGF21 undergoes upregulation in the diabetic environment, exerting both metabolic regulation and immunomodulatory effects. Consequently, it holds potential as a valuable marker for the early diagnosis and prognosis of DKD (**Figure 3A**). A cohort study in China, encompassing 312 patients with T2DM who had their baseline FGF21 levels measured and were subsequently followed for 6 months, defined renal endpoint events as a 30% decrease in eGFR or worsening categories of albuminuria. The findings revealed a correlation between FGF-21 levels and the risks of renal events in a broad-spectrum of Chinese T2DM subjects (70). In a prospective observational study examining the association of soluble tumor necrosis factor receptor type 1 (sTNFR1), FGF-21, endocan, N-terminal pro-brain natriuretic peptide (NT-pro-BNP), and renal outcomes in patients with or without clinical signs of DKD, both sTNFR1 and FGF-21 levels in patients with T2DM were linked to renal outcomes. The combination of these markers demonstrated improved predictability (71). A meta-analysis involving 28 studies with 19,348 participants indicated that a high serum FGF21 level may predict the incidence of chronic kidney disease (CKD) and renal outcomes in patients with T2DM (72). A study involving 1136 Chinese T2DM patients revealed that serum FGF21 levels increased progressively with eGFR category. In a subset comprising 559 individuals with normoalbuminuria and baseline eGFR ≥ 60 mL/min/1.73 m^2, serum FGF21 continued to be a reliable indicator of eGFR decline. Elevated serum FGF21 levels were proposed as a useful biomarker for predicting the progression of renal disease, particularly in the early stages of DKD (73). Another cross-sectional study with 130 individuals demonstrated that serum FGF21 was independently associated with microalbuminuria in patients with T2DM (74). An intriguing prospective cohort study in Singapore, involving 1700 Asian people with T2DM and a mean follow-up of 6.3 years, found that in women with T2DM, plasma FGF21 levels independently predicted the risk of progression to end-stage renal disease. Further research is needed to comprehend the pathophysiological connections between FGF21, sex, and renal progression (75).

## 5 FGF21 is a potential target for DKD therapy

FGF21 plays a crucial role in regulating both energy metabolism and innate immunity, making it a potential target for therapeutic interventions in DKD (**Figure 3B**). Based on the kidney disease: improving global outcomes (KDIGO) 2022 clinical practice guideline for diabetes management in chronic kidney disease, the treatment of DKD involves both life management and medication (76). Diet and exercise management, and multiple guideline-recommended hypoglycemic agents are associated with FGF21.An experimental study revealed that fasting-induced FGF21 signaling activates hepatic autophagy and lipid degradation through the JMJD3 histone demethylase (66). In humans, circulating FGF21 levels experienced a significant surge after 28 days on a low-protein (LP) diet, establishing FGF21 as an endocrine signal for protein restriction, coordinating metabolism and growth during reduced protein intake (77). Fasting, protein restriction, and specific reductions in essential amino acid levels impact FGF21 activity, promoting healthy longevity (78). Muscle exercise stimulates FGF21 production in muscles, subsequently secreted into circulation to induce lipophagy in the liver through an AMPK-dependent pathway (79). Through PPARγ-mediated transcriptional activation, exercise induces adipose expression of FGFR1 and β-klotho, sensitizing FGF21 actions in adipose tissues to send humoral signals for multi-organ crosstalk, thereby maintaining metabolic homeostasis (80). A systematic review, involving 376 participants from 10 studies, demonstrated a significant increase in FGF-21 levels after exercise compared to no exercise (81). Long-term aerobic exercise has been observed to lower human FGF21 levels and is associated with an improvement in FGF21 resistance (34).

SGLT2 inhibitors (SGLT2i) have emerged as the primary choice  for DKD with cardiorenal protective effects. Studies indicate that the SGLT2 inhibitor canagliflozin induces transcriptional reprogramming, activating catabolic pathways, increasing fatty acid oxidation, reducing hepatic steatosis and diacylglycerol content, and elevating hepatic and plasma FGF21 levels (82). Furthermore, empagliflozin shifts energy metabolism towards fat utilization, increases phosphorylation levels of AMP-activated protein kinase and acetyl-CoA carboxylase in skeletal muscle, and enhances liver and plasma FGF21 levels (83). The mechanism behind SGLT2 inhibitors preserving renal function in T2DM while promoting ketogenesis may be attributed to the activation of SIRT1/PGC-1α/FGF21 (84). A sub-study of the DAPA-VO2 investigation revealed that, within the cohort of patients experiencing stable heart failure with reduced ejection fraction (HFrEF), dapagliflozin initiated a temporary elevation in klotho levels. The author speculates that the observed increase in klotho may potentially signify an improvement in proximal tubular function mediated through the reduction of oxidative stress and inflammation within the renal tubules. This improvement might lead to a decreased resistance to FGF-23 (85). Another class of hypoglycemic agents with weight-loss effects, glucagon-like peptide 1 (GLP-1) receptor agonist, is also strongly associated with FGF21. Experimental studies have demonstrated that hepatic FGF21 is essential for liraglutide to effectively reduce body weight and enhance hepatic lipid homeostasis (86). In a clinical study, it was shown that exenatide could aid patients in improving glycemic levels, inflammatory biomarkers, and urine albumin concentration. The effects of exenatide may be partially mediated by FGF21 (87). The traditional glucose-lowering drug, metformin, remains the first-line agent for treating DKD. A clinical study suggests that metformin suppresses circulating fibroblast activation protein activity, upregulates the expression of FGFR1c and β-klotho, thereby increasing FGF21 signaling in adipose tissue and improving peripheral FGF21 sensitivity (88). Additionally, insulin also stimulates FGF21 expression; insulin-induced expression of muscle FGF21 is strongly correlated with an increase in serum FGF21, and this response appears to be intact in T2DM (89). The action of the peroxisome proliferator-activated receptor gamma (PPARγ) agonist rosiglitazone is also reliant on FGF21. FGF21-KO mice do not respond to both the beneficial insulin-sensitizing effects and the adverse side effects of weight gain and edema associated with rosiglitazone (90).

In addition to existing medications, several novel FGF21-based drugs and therapies have demonstrated therapeutic potential for diabetic kidney disease (DKD). The natural form of FGF21 poses challenges for biopharmaceutical applications due to its short half-life, tendency to aggregate in soluble preparations, and susceptibility to protein hydrolysis. As a result, biopharmaceutical technology has undergone significant advancements to extend the duration of action of FGF21 (91). Preclinical studies showed that PEGylated recombinant human FGF21(PEG-rhFGF21) significantly lowered lipid levels in the kidney, decreased urine albumin/creatinine ratio (ACR) and improved mesangial expansion in db/db and DIO mice (92). A randomized, placebo-controlled, double-blind proof-of-concept trial conducted in patients with obesity and T2DM revealed that LY2405319 (LY), an investigational FGF21 variant deemed suitable for early-phase clinical development, demonstrated significant improvements in dyslipidemia. Positive outcomes were observed in terms of increased adiponectin levels, reduced fasting insulin, and decreased body weight. However, it should be noted that only a trend toward glucose lowering was observed (93). LLF580 represents a novel genetically engineered variant of human FGF21, distinguished by its stabilization through the introduction of a disulfide bond and fusion at its N-terminus to the human IgG (subclass IgG1) Fc domain. Generally considered safe, LLF580 exhibits favorable effects on biomarkers associated with lipids, liver fat, and liver injury. In comparison to a placebo, insulin levels, C-peptide levels, and insulin resistance measured by the homeostatic model were all lower, while adiponectin levels were higher during LLF580 treatment. However, it is noteworthy that fasting glucose and glycated hemoglobin remained unchanged (94). The results of a phase II clinical trial indicate that Pegbelfermin (BMS-986036), a PEGylated FGF21 analog, did not exert an impact on HbA1c concentrations. However, weekly (QW) and higher daily doses demonstrated an association with improved metabolic parameters and fibrosis biomarkers in patients with obesity and T2DM who were predisposed to fatty liver (95).

Although the aforementioned FGF21-based new drugs have shown a trend towards improving metabolism, they did not impact HbA1c concentrations in clinical studies. Considering the elevated serum FGF21 levels in metabolic disorders such as T2DM and obesity, and the potential association with FGF21 resistance, artificially increasing the levels of FGF21 (or FGF21 analogs) may not necessarily enhance their actual biological effects in such states of metabolic disruption. The formulation of FGF21 may offer enhanced benefits when combined with other drugs targeting glycemic control and weight loss. In db/db mice, the combined administration of subtherapeutic doses of FGF21 and insulin maintains blood glucose levels for at least 24 hours, inhibits weight gain, and significantly improves lipid parameters. These results indicate that insulin renders FGF21 more sensitive in regulating glucose and lipid metabolism (96). The combined treatment of FGF21 with insulin further improves various parameters, including blood glucose, HbA1c, oral glucose tolerance tests (OGTT), renal function, liver function, blood lipid levels, histopathological alterations, oxidative stress (OS), and advanced glycation end-products (AGEs) in mice with diabetic kidney disease (DKD), compared to insulin or FGF21 administered alone (56). A novel biological agent, which fuses GLP-1 to FGF21 using an elastin-like polypeptide linker acting as a sustained release module with zero-order drug release, has been developed. Administering this dual agonist once a week to diabetic mice results in significant weight loss and improved glycemic control, effects not observed when either agonist is used alone. Moreover, the dual-agonist formulation surpasses the efficacy of a GLP-1/FGF21 combination, underscoring the value of integrating two structurally different peptides into a single, multipurpose molecule (97). Additionally, a novel GLP-1/FGF21 dual agonist has demonstrated superior weight loss effects compared to GLP-1 or FGF21 administered individually (98).

## 6 Summary and prospect

DKD is highly prevalent and poses significant harm, with ongoing opportunities for advancements in its diagnosis and treatment. The progression of DKD is associated with a variety of metabolic dysregulations, and glucose and lipid metabolites in the DM setting can directly damage the kidneys while further exacerbating metabolic disturbances. Innate immunity plays an important role in DKD and microinflammation can persistently exacerbate kidney damage. At the same time, metabolic disorders can affect innate immune cell metabolism, signaling and the release of various cytokines. Crosstalk between energy metabolism and innate immunity is one of the main targets of DKD. FGF21, induced by factors such as diet, exercise, cold exposure, and metabolic disorders, is significantly upregulated in diabetic environments. FGF21 forms a complex with FGFR1 and β-klotho, activating AMPK signaling in target tissues and promoting adiponectin expression. The multifaceted roles of FGF21 include the regulation of metabolism by altering brain nutrient preferences, enhancing glucose uptake in adipose tissue, increasing urinary glucose excretion, and promoting insulin secretion and sensitivity. Moreover, FGF21 directly regulates innate immune signals and pathways, while also indirectly influencing innate immunity through mechanisms such as OS, autophagy, and ferroptosis. This dual action enables FGF21 to safeguard renal endothelial cells and impede the progression of DKD. Functioning as a bridge between energy metabolism and innate immunity, FGF21 emerges as a potential marker for both the diagnosis and prognosis of DKD, presenting itself as a promising therapeutic target. Several clinical studies have demonstrated associations between FGF21, DKD proteinuria, and eGFR. Additionally, various current treatments for DKD, including dietary interventions and exercise, exhibit connections with FGF21. The promising metabolic modulation effects of numerous novel FGF21-based drugs further underscore the potential of FGF21 as a pivotal player in DKD management and therapy.

Despite the significant potential of FGF21 in DKD, several challenges persist that warrant attention. Firstly, there is an insufficient body of basic research on the interactions between FGF21 and DKD. While FGF21 is known for its clear metabolic regulation and innate immunomodulatory effects, the specific mechanisms underlying its actions in the context of DKD remain unclear and warrant further investigation. Secondly, many clinical studies examining FGF21 and DKD are characterized by single-center designs and small sample sizes. Consequently, the value of FGF21 in the diagnosis and prognosis of DKD needs to be validated through larger, multi-center studies with diverse patient populations. Robust and comprehensive clinical evidence is essential to establish FGF21 as a reliable biomarker for DKD. Finally, several new drugs developed based on FGF21 have demonstrated metabolic benefits, but their impact on glycemic improvement and innate immunity modulation remains unclear. These drugs have not been extensively studied in clinical trials specifically focused on DKD. The potential of combining novel hypoglycemic agents with FGF21 for enhanced therapeutic effects is promising, but further research is essential to understand the comprehensive impact of these interventions in the context of DKD. Addressing these research gaps will contribute to a more thorough understanding of FGF21’s role in DKD and may pave the way for more effective therapeutic strategies.

## Author contributions

YL: Writing – original draft. QC: Writing – original draft. YC: Writing – original draft. JH: Writing – original draft. JY: Writing – original draft. ZC: Writing – review & editing. JZ: Writing – review & editing.
